# Supplementary material for: Are carbon emissions trading and green financial instruments synergistic? -Comprehensive quantitative research based on content analysis
Source: PLoS One. 2024 Mar 7;19(3):e0298601. doi: 10.1371/journal.pone.0298601 (PMC10919601; doi:10.1371/journal.pone.0298601)
Supplement: S1 Data — (ZIP) [file pone.0298601.s001.zip › Data/地级市Z_F工作报告.zip!/║╙─╧/╓ú╓▌╩╨/└┤╫╘╣1⁄2╓┌║┼í╛┬φ┐╦╩2╛▌═°í┐_2008.docx]

现在，我代表市人民政府向大会作工作报告，请予审议，并请各位政协委员提出意见。

　　一、２００７年政府工作回顾

　　２００７年，市人民政府在省委、省政府和市委的正确领导下，团结带领全市人民，高举中国特色社会主义伟大旗帜，坚持以邓小平理论和“三个代表”重要思想为指导，认真学习贯彻党的十七大精神，全面落实科学发展观，按照“加快发展、维护稳定、为民谋利”的总体要求，开拓进取，扎实工作，较好地完成了市十二届人大四次会议和跨越式发展“三年行动计划”确定的年度目标任务，实现了经济社会又好又快发展。全年实现生产总值２４２１．２亿元，增长１５．６％；人均生产总值达到３３１６９元，增长１４％；地方财政收入达到２７７．６亿元，增长３７．２％；全社会固定资产投资完成１３６７．３亿元，增长３２．５％；利用域外资金达到４１３亿元，增长６６．３％；社会消费品零售总额完成９７８．７亿元，增长１９％；城镇居民人均可支配收入达到１３６９２元，农民人均纯收入达到６５９４元，分别增长１５．８％和１８．６％。

　　（一）工业质量和效益进一步提高。大力实施扶优扶强、产业园区建设和科技自主创新工程，工业经济实现了持续快速健康发展。全部工业完成增加值１１６６．９亿元，增长２０．８％。其中，全市规模以上工业企业完成增加值９２３．８亿元，增长２４．３％；实现产品销售收入３２６３．３亿元，增长４０．２％；实现利税５７９．９亿元，增长５７．７％。全市销售收入超亿元企业新增２３６户，达到７５９户，宇通公司、中铝河南分公司、河南中烟公司、郑煤集团、豫联集团、郑州供电公司销售收入超百亿元。全年完成工业投入５３２．６亿元，增长６３．２％。中铝公司郑州铝加工厂年产１２万吨高精度铝板带材、天津顶新公司康师傅食品基地三期等一批重点项目竣工投产，郑州日产年产１．３万辆ＭＰＶ多用途乘用车和１．２万辆ＳＵＶ乘用车项目新车下线，郑州东５００千伏输变电工程顺利开工，燃气电站２×３９万千瓦机组等一批电源项目建成投产，全市全年新增装机容量１６９万千瓦，总容量达到６０６万千瓦。重点企业战略重组迈出实质性步伐，省投资公司重组中原制药厂；郑州轻汽与海马集团、郑缆集团与中科英华成功实现了战略重组；棉纺企业战略重组稳步推进，形成新的生产能力，企业逐步走出低谷。海力特、威科姆、方欣生物等一批高新技术企业快速成长。全市高新技术产业完成增加值３２３．４亿元，增长３３．７％；全年专利申请５２１４件，增长５５．１％。我市荣获全国科技进步先进城市称号。

　　（二）“三农”工作稳步推进。不断加大农业投入，市财政全年用于“三农”的总支出达到３６．６亿元，增长３２．７％。继续落实支农惠农政策，共发放种粮补贴、良种补贴、农资综合直补等各项资金１．９亿元，农民的生产积极性进一步提高。粮食生产全面丰收，总产量达到１７１．１万吨，是历史上第二个高产年。农业产业化经营稳步推进，全市已发展农业产业化经营组织９５９个，市级以上重点龙头企业２１６家。农业对外开放水平不断提高，全市农业出口创汇企业达到３１家，完成出口创汇６７５４万美元，增长１９．５％。农村生产生活条件不断改善，全年投资１．４亿元解决３６．１万人的安全饮水问题，新建集雨水窖１万个，新建沼气池５万座，改扩建农村公路１０５６公里，解决了贫困地区１万人的温饱问题。继续实施“百村示范、千村整治”工程，新农村建设成效明显。确定的１０４个示范村都制定了产业发展规划，有９４个村制定了村庄建设规划，“五通”、“三有”建设项目基本完成。２０个重点示范村建设基本达到标准，整治村的村容村貌发生较大变化。

　　（三）服务业发展实现新突破。壮大提升批零贸易、住宿餐饮、交通运输等传统服务业，加快发展现代物流、会展、金融、旅游等现代服务业。鼓励丹尼斯百货、大商新玛特公司等一批商业企业做大做强，郑州中原国际小商品城、大上海城等商业项目建设顺利，１２个专业批发市场辐射力进一步增强。加快特色商业街区建设，鼓励促进夜间消费。“万村千乡市场工程”顺利推进，全市已建成达到国家标准的农家超市１１００多家。加快物流业发展，编制完成了《郑州航空港概念性总体规划》和《中原国际物流园区控制性详细规划》，郑州铁路集装箱中心站、郑州国家干线公路物流港建设速度加快，积极支持豫鑫物流、市交运集团和长通物流等企业做大做强。加快会展业发展，成功举办了第二届中部投资贸易博览会、首届中国（郑州）印刷包装产品博览会、第３３届全国制药机械博览会、第６２届全国汽车配件交易会和全国农机产品订货交易会等一批大型展会，全年展览面积突破１００万平方米。区域性金融中心建设稳步推进。认真落实《郑州区域性金融中心建设规划纲要》，出台了《关于鼓励外资金融机构落户郑州的意见》，汇丰银行、民生银行已获准在我市设立分支机构。银行、证券、保险、期货、信托等金融产业快速发展，年末全市金融机构各项存款余额达到３９３８．２亿元，各项贷款余额２９３９．９亿元，分别比上年末增长１０．６％和８％。郑州商品交易所交易量达１．８６亿手，交易额达５．９万亿元，分别增长１０１％和８６％。房地产业平稳发展，全年新开工商品房１３９９．９万平方米，增长１９％；房地产业完成增加值８６．１亿元，增长１４．８％。大力发展文化旅游产业，商城遗址公园、郑州歌舞剧院等一批重点文化建设工程进展顺利，嵩山历史建筑群申报世界文化遗产工作稳步推进，嵩山少林景区被评为国家首批５Ａ级景区，黄帝故里景区完成了改扩建工程，炎黄二帝巨塑正式落成，丁亥年黄帝故里拜祖大典成功举办，《禅宗少林?音乐大典》和《快乐星球》受到广泛赞誉，大型舞剧《云水洛神》创作进展顺利。全年接待海内外游客２９３７．２万人次，增长２５．７％；实现旅游总收入２８５．８亿元，增长２４．５％。

　（四）跨越式发展八项重点工程进展顺利。积极协调解决项目用地、环评审批和资金筹措等方面的问题，强力推进重大项目建设。扶优扶强企业完成销售收入１０２０亿元，增长３８％。全市重点产业园区完成工业增加值６２２．１亿元，实现销售收入２００９．５亿元，分别增长３３．８％和３９．３％；全市销售收入超百亿元、超５０亿元的重点工业园区新增７个，总数达到１３个。科技自主创新工程２９家企业共完成投资１２．８亿元；销售收入达到４６．２亿元，增长３５．７％。新农村建设工程扎实推进，１０４个示范村新增投资５亿元，２０个重点示范村建设成效明显。城市建设和管理工程２５个重点项目全部开工，完成投资１６５亿元以上。交通枢纽和物流工程３３个项目已开工１４个，竣工６个。文化建设工程３０个项目进展顺利。生态建设工程各项年度目标已圆满完成。郑汴产业带、郑州轻汽改制重组及扩建等２０个重大发展项目加快推进，全年完成投资２９．６亿元。

　　（五）城市建设和管理迈出新步伐。加快城市总体规划和土地利用总体规划的修编工作，完成了《郑州市城市综合交通近期建设规划》、《郑州市城市生态水系规划》等一系列重大规划的编制工作，并开始组织实施。深入开展城市管理年活动，中心城区功能不断完善。中心城区建设改造资金达到７０亿元，重点对道路交通、园林绿化、城市水系、户外广告等９个方面进行了专项整治。进一步完善路网结构，中原西路、郑邙路、秦岭路等１７项道路工程竣工，“一站一桥一路”工程扎实推进。城中村改造政策进一步完善，拆迁和建设步伐明显加快。郑东新区“五年成规模”的目标基本实现。全年新增投资１３０亿元，累计完成投资５０６．４亿元；新引进项目３６个，新开工项目６５个，累计开工项目３８１个；开发面积达到５０平方公里，在建和建成房屋面积达到１７００万平方米，入驻人口突破２２万人。中央商务区建设进一步加快，功能不断完善，内环３０栋高层全部竣工，外环２７栋高层顺利开工，河南省艺术中心建成并投入使用，会展宾馆正进行基础施工，商业步行街项目加速推进。中国农业银行河南省分行、新华人寿保险河南分公司等１４家金融机构和一批企业总部正式入驻。龙湖南区、商住物流区入驻项目建设进展顺利。加快卫星城和小城镇建设步伐，各项设施和功能不断完善。全市城镇化率达到６１．３％。郑汴一体化进程不断加快。《郑汴产业带总体规划（２００６－２０２０）》正式通过，郑汴产业带控制性详细规划编制完成，有关专项规划和设计全面启动，已确定入驻项目３６个，总投资达１９２亿元。

　　（六）改革开放不断深入。国有企业改制取得明显进展，白鸽股份资产重组和股权分置顺利完成，紫荆山百货大楼由新加坡时计宝投资有限公司成功重组，金牛集团、飞马集团等企业的破产工作基本完成。进一步放宽市场准入，不断优化发展环境，非公有制经济快速发展，非公有制经济增加值占全市生产总值的比重达到５８％，民间投资占全市城镇固定资产投资的比重达到６９．１％。农村综合改革深入推进，粮食流通和供销体制改革取得新进展。投融资体制改革步伐加快，市国投公司、城建集团、住宅投资公司、交建投公司和文化产业投资公司相继成立运营。企业上市融资成效明显，又有恒星科技、耕生耐材、恒发能源、鑫苑置业、中阀科技等５家企业在境外上市，郑州燃气在香港转主板上市，全市上市企业总数达到１７个，全年通过证券市场融资３９．８亿元。出口和利用外资取得新突破。全市直接进出口总额达到３１．６亿美元，增长２４．７％，其中，完成出口总额２１．７亿美元，增长２０．５％。实际利用外资达到１０亿美元，增长６３．１％；引进境内域外资金３３９．２亿元，增长６９．５％。

　　（七）生态环境建设成效明显。出台了《郑州市节能减排实施方案》，加快重点领域、重点区域和重点行业的节能减排工作，市财政投入１０４９万元专项扶持资金支持节能减排。全年万元生产总值能耗下降超过４％，全市化学需氧量和二氧化硫排放总量较上年分别削减４．８％和４．１％。矿产资源整合工作取得新进展，全市煤矿数量由５０１家整合为２２３家，７８家铝土矿整合为２２家。“五小”企业关停力度不断加大，土地“三项整治”深入推进，淘汰落后产能步伐加快。环境质量得到改善，市区空气质量二级以上天数达到３１３天。加强生态建设，生态林业建设完成投资４．７亿元，新增造林２２．２万亩，其中，森林生态城建设新增造林１７．６万亩，建成区绿化覆盖率达到３３．８％，荣获全国绿化模范城市称号。加强城市生态水系的规划和建设，须水河、潮河、索须河上游段河道治理和东风渠引黄供水补源灌溉工程全部完工。马头岗污水处理厂建成投入使用，王新庄污水处理厂脱氮改造工程进展顺利，市垃圾综合处理场建成运行，６个县（市）污水处理厂和垃圾处理场全部建成并投入运营。大力开展创建国家环保模范城市工作，３２项考核指标基本达标。

　　（八）民生继续得到改善。进一步调整财政支出结构，加大对民生问题的投入，向人民群众承诺的“十件实事”全部完成。全年用于解决民生问题的财政支出达到８９．５亿元，增长３８．５％，占一般预算支出的３７．２％。就业再就业工作成效明显，实现城镇就业１２．６万人，农村劳动力转移就业１２．７万人，对农村劳动力进行引导性培训１３．２万人次。全市企业职工养老保险新增参保人数达到１０．１万人；城镇居民基本医疗保险工作全面推进，参保人数达到６５万人；农村新型合作医疗制度全面推行，参合人数达到３８２万人，参合率达到９５．５％。城乡低保标准全面提高。针对副食品价格上涨的形势，动用价格调节基金，对城镇低保户、在校大学生、离退休职工和困难户发放了补贴。继续加大教育事业投入。在全省率先免除农村义务教育阶段学生的课本费和作业本费，全年拨付“两免一补”资金１．６亿元；为全市农村义务教育阶段中小学更新配备课桌椅２１．９６万套。高度重视卫生事业发展。市、县两级投入９０００万元完成了乡镇卫生院建设改造任务，配置了Ｘ光机、心电图仪等一批重点医疗设备。为全市１．４万例白内障患者免费实施了复明手术。新建改建敬老院２８所，农村五保户集中供养率提高到４１％。加强社区建设，新建社区文化中心１２１个、社区卫生服务中心３２所。不断完善社会保障性住房的政策体系，着力改善低收入群体的住房条件。全年新开工经济适用住房１０９．３万平方米，竣工７６．１万平方米，市区廉租住房覆盖率达到符合条件“双困家庭”的１００％。实施食品药品放心工程和农产品市场准入制度，有效保证了人民群众饮食、用药安全。人口和计划生育工作成效明显，农村人口和计划生育工作走在了全国前列，流动人口计划生育管理和服务得到加强，低生育水平继续稳定。

　　同时，民主法制建设和社会稳定工作不断加强。自觉接受人大及其常委会和政协监督。高度重视政府法制工作，全年提请审议地方性法规２件，制定规章１３件。认真落实党风廉政建设责任制，政府廉政建设和反腐败工作取得新成效。严格落实安全生产责任制，对重点行业、重点领域进行全面检查和专项整治，安全生产形势总体平稳。深入开展平安建设，大力开展“四严一创”活动，依法打击各类违法犯罪，“五五”普法深入开展，信访工作机制不断创新，应急管理体制不断完善，社会治安持续稳定。加强军政军民团结，我市连续五次荣获全国双拥模范城市称号。

　　广播电视、统计、审计、物价、外事、侨务、对台事务、民族宗教、社会科学、地方志、档案管理、人防、气象和地震监测等工作都取得了新的成绩。

　　回顾一年来的工作，我们深刻体会到，我市经济社会能够实现又好又快发展，得益于认真坚持和把握了以下几个方面：一是以科学发展观为指导继续实施跨越式发展战略。以重点工程和重大发展项目为抓手，推动了一批重大基础产业、交通、城市建设、生态环境和社会事业项目建设，为我市今后一个时期的发展抢得了先机，赢得了主动。二是抓住我市经济社会发展的薄弱环节实施重点突破。在第三产业发展、城区经济发展等方面采取了一系列重大措施，使我市经济发展更加健康、后劲更加充足。三是超前谋划、科学规划郑州未来的发展战略。立足当前，着力谋划打基础、管长远的重大布局。加快推进郑东新区、郑汴产业带和郑州航空港区的开发建设，进一步拓展发展空间；强力实施森林生态城和城市生态水系建设，推进一批环保基础设施建设，打造良好人居环境，使我市在未来的竞争中站位高、定位准，优势更加凸显。四是更加注重保障和改善民生。坚持以人为本，在经济发展的基础上，更加注重社会建设，扩大公共服务，完善社会管理，千方百计解决关系人民群众切身利益的问题，形成了全市上下共建共享的和谐局面。

　　各位代表，上述成绩的取得，是省委、省政府和市委正确领导的结果，是市人大、市政协监督支持的结果，是全市广大干部群众共同奋斗的结果。在此，我代表市人民政府，向工作在全市各条战线的广大工人、农民、知识分子、干部、驻郑部队、武警官兵、公安民警以及各界人士致以崇高的敬意！向关心、支持郑州发展的香港特别行政区同胞、澳门特别行政区同胞、台湾同胞、海外侨胞和国际友人表示衷心的感谢！

　　各位代表，尽管我市经济社会发展取得了一些成绩，但我们也清醒地认识到发展中存在的矛盾、困难和问题，主要表现在：消费和出口带动作用不强，第三产业增长相对缓慢，自主创新能力较弱，结构调整和转变发展方式任务比较艰巨；高耗能、资源型工业比重较大，节能减排形势不容乐观；土地、资金、环境等约束加剧，发展的制约因素增多；市场物价持续高位运行，低收入群体生活受到一定影响；劳动就业、社会保障、教育、医疗卫生、住房等关系人民群众切身利益的问题解决得还不够好；一些地方和部门工作人员服务意识还不够强，效率还不够高，发展环境还需要进一步优化。我们一定要保持清醒头脑，增强忧患意识，沉着应对，克难攻坚，扎实做好政府各项工作。

　　二、２００８年政府工作的基本要求和主要目标

　　今年是全面贯彻落实党的十七大精神的第一年，是我国改革开放３０周年，是“十一五”发展的关键之年，也是本届政府任期的最后一年。做好今年的政府工作，对于全面完成市十二届人大一次会议确定的各项目标任务、保持经济社会跨越式发展的良好势头、夺取全面建设小康社会新胜利意义十分重大。今年政府工作的基本要求是：全面贯彻党的十七大精神，高举中国特色社会主义伟大旗帜，以邓小平理论和“三个代表”重要思想为指导，坚持科学发展观，认真落实宏观调控政策，按照“加快发展、维护稳定、为民谋利”的总体要求，围绕建设全国区域性中心城市目标，加快经济社会跨越式发展步伐，大力推进工业化、城镇化和农业现代化，积极推进改革开放和自主创新，着力优化经济结构，转变发展方式，提高经济增长质量，切实加强节能减排和生态环境保护，更加重视改善民生和促进社会和谐，加快建设文化郑州、生态郑州、平安郑州、和谐郑州。

　　今年我市经济社会发展的主要目标是：生产总值增长１３％，其中，第一、二、三产业增加值分别增长３％、１４％和１３％；地方财政收入增长１５％；全社会固定资产投资增长２０％，其中，城镇投资增长２５％；社会消费品零售总额增长１５％；实际利用外资额增长３０％；居民消费价格涨幅略低于上年实际水平；城镇居民人均可支配收入增长９％，农民人均纯收入增长１０％；人口自然增长率控制在６％。以下；城镇登记失业率控制在４％以内，城镇新增就业１２万人；城镇化率提高１个百分点；万元生产总值能耗降低５．４％，化学需氧量削减率达到４．５％，二氧化硫削减率达到５％。

　　为全面完成上述目标任务，我们必须进一步解放思想，转变观念，认真把握好以下几个重要方面，切实把经济社会发展转到科学发展的轨道。

　　一是坚持稳中求进、好字优先，着力推进“三个转变”。经济发展由主要依靠投资拉动向依靠消费、投资、出口协调拉动转变，由主要依靠第二产业带动向依靠第一、第二、第三产业协同带动转变，由主要依靠增加物质资源消耗向主要依靠科技进步、劳动者素质提高和管理创新转变，以汽车制造、现代物流、文化旅游等条件成熟、关联度高、带动力强的产业为重点，全力推进产业结构优化升级，努力做到速度与结构、质量、效益相统一，经济发展与人口、资源、环境相协调，实现又好又快的发展。

　　二是统筹城乡和区域协调发展，着力优化生产力布局。按照形成城乡经济社会发展一体化新格局的要求，在发展规划、产业布局、基础设施、公共服务、劳动就业和社会管理等方面加强城乡统筹，促进城乡市场开放统一、生产要素有序流动、经济社会协调发展，努力实现城乡共同繁荣。在不断发展壮大县域经济的同时，进一步加快城区经济发展步伐，努力形成县域经济和城区经济互相促进、共同发展的良好局面。

　　三是强化中心城市意识，着力用现代化理念构建大郑州。按照郑州在中原城市群的功能定位，进一步增强中心意识，完善中心功能，壮大中心产业，发挥好中心城市带动作用。加强规划引导，高标准编制全国区域性中心城市建设规划，抓紧完成城市总体规划修编的报批工作，制定和完善区域性控制规划，促进城市规模结构合理化、布局集群化、土地利用集约化。加强城市基础设施建设和公共服务体系建设，增强城市综合承载能力。

　　四是坚持资源节约和环境保护，着力建设生态文明。把推进现代化与建设生态文明有机统一起来，把建设资源节约型、环境友好型社会放在工业化、现代化发展战略的突出位置，加快形成节约能源资源和保护生态环境的产业结构、发展方式、消费模式。

　　五是坚持以人为本，着力改善民生。大力发展就业、教育、住房、社会保障、医疗卫生等各项社会事业，切实解决群众最关心、最直接、最现实的利益问题，使全市人民共享发展成果。

　　三、加快经济结构调整和产业优化升级

　　以结构调整为主线，着力转变经济发展方式，进一步提高我市经济的整体素质。

　　（一）优化工业结构，提升产业竞争力。继续把扶优扶强和产业园区建设作为工业经济的着力点，以项目为载体，深入开展工业结构调整提速年活动，进一步提升工业竞争力。一是继续做大做强重点企业。全年工业投入力争达到７００亿元以上。进一步支持我市省定１３户重点工业企业和１３户高成长型企业的发展，筛选确定我市１００户重点工业企业和５０户高成长型企业，制定政策进行重点扶持，尽快培育一批跨地区、跨行业、跨所有制的大型企业集团，力争全年销售收入超百亿元企业新增２家以上。力争开工建设大成生物质化工、郑州煤化工、郑州日产年产１０万辆整车、海马（郑州）汽车、宇通集团郑汴产业带零部件产业基地、郑纺机、郑煤机高端产业园等一批项目；加快中铝河南分公司氧化铝提产增效项目的建设进度；继续加快赵家寨年产３００万吨煤矿建设，力争年内主体工程完工。加快产业集聚，加强３３个重点产业园区的规划建设，尽快形成一批特色产业园区。到年底，重点产业园区工业增加值达到７５０亿元，实现销售收入２５００亿元。二是提高企业自主创新能力。支持企业建立研发中心，列入省、市百户重点工业企业和５０户高成长型企业都要建立研发中心，争取新增国家级研发中心１家以上、省级研发中心３５家以上。强化政府投入的导向作用，鼓励企业加大科研投入，努力将企业研发投入占销售收入的比例提高到３％以上。加大对科技创新的扶持，争取在电子信息、生物制药、光机电一体化、新材料等领域实现重大突破。支持企业加强现代化和信息化建设，大力推进信息化与工业化的融合。实施品牌发展战略，力争再创一批国家级名牌产品和在国际上有影响的知名品牌。做好知识产权保护工作。三是加快推进企业战略重组和上市步伐。加快构建国有资本退出和外资、民资、社会法人资本进入的新机制，推进企业产权多元化。认真落实推进企业上市的有关政策，支持已经上市的企业增发新股、配股和发行可转换债券。四是高度重视企业的经营管理。进一步加强对企业的宏观管理和指导，落实扶持政策，推进管理创新，狠抓企业扭亏增盈。全力做好煤电油运保障工作。

　　（二）大力发展服务业，提高现代服务业发展水平。积极推进第三产业优化升级，着力发展壮大现代服务业。一是加快发展辐射带动能力强的商贸流通业和专业批发市场。进一步完善市级商业中心、区级商业中心和社区商业网点三个层面的商贸流通网络。推动二七商圈、碧沙岗商圈、紫荆商圈实施产业升级，培育壮大郑东新区ＣＢＤ商圈、火车站西出站口商圈、郑州铁路新客运站商圈。制定市场管理条例，大力推进区域性专业商品交易市场建设，加快社区商业发展。积极发展连锁经营、物流配送、电子商务等新型业态。二是加快发展物流业。采取国际招标的办法，编制完善现代物流业发展规划，全面提升物流业发展档次。加快圃田、航空港两个物流园区和１１个现代物流中心建设，积极推进郑州铁路集装箱中心站、郑州国家干线公路物流港和中南邮政物流集散中心建设，抓好河南进口物资保税中心、长通物流中心等一批在建物流项目，加快粮油、农资、医药、出版、汽车及零部件等重点行业物流发展。扶持重点物流企业发展，争取培育１家５Ａ级物流企业、引进１家国际著名物流企业。三是加快发展会展业。积极申办国内外大型展会，举办好国际农产品交易会、第１０届亚洲艺术节、第１８届全国图书交易博览会等一批重点展会；大力培育我市自主品牌展会，办好第１４届郑州全国商品交易会和中国郑州纺织品服装博览会。力争全年展会数量达到１００个，展览面积突破１２０万平方米。四是加快发展金融业。加大国内外知名金融机构引进力度，积极支持东亚银行等金融机构在我市设立分支机构。鼓励创新类和规范类证券公司、期货机构和保险机构在我市设立分支机构。支持郑州商品交易所做大做强。加快培育资本市场，增强企业融资能力。五是加快发展服务外包产业。抓紧研究制定服务外包产业发展专项规划，重点扶持信息技术外包和业务流程外包，加快申报国家服务外包基地城市。六是注重消费对经济增长的拉动作用。积极培育节日消费、夜间消费、午间消费，扩大通信、文化、旅游、体育等新的消费热点。实施“家电下乡”工程，对农民购买规定类家电予以补贴。

　　（三）继续抓好八项重点工程，强力推进重大项目建设。坚持跨越式发展工作例会制度和重大发展项目工作推进机制，坚持解决问题台账制度、督查考核制度和现场办公制度。落实重点工程项目领导分包联系责任制、联席办公会、综合审批制度，加大重点工程、重大项目推进力度，确保跨越式发展“三年行动计划”各项目标任务的圆满完成。同时，及早谋划、储备、跟踪一批重大项目，做好实施新一轮跨越式发展行动计划的准备工作。以项目为支撑，对初步排定的２００８～２０１２年３６４个重大产业项目进行确认，按进度梳理排队，实行一个项目、一套班子、一抓到底的“三个一”工作推进机制，逐一明确责任主体，组建项目班子，抓好一批具有重大战略意义的项目，切实增强我市经济社会发展的后劲和活力。

　　（四）统筹区域经济协调发展，进一步提升城市综合竞争力。城区经济和县域经济是我市经济社会跨越式发展的“双重引擎”，要统筹兼顾，全面推进。一是加快发展城区经济。科学制定城区经济发展规划，明晰城区经济功能定位。以提升产业层次为重点，优化城区产业布局。加快发展现代物流、信息咨询、中介服务、会展、金融保险、服务外包、创意产业等新兴服务业。大力发展楼宇经济、总部经济。以产业园区为载体，不断提升都市工业的质量和水平。继续支持高新区、经开区和出口加工区加快发展。二是继续发展壮大县域经济。进一步创新发展观念和发展模式，以改革创新为动力，以工业经济为主导，以农业产业化为载体，着力发展特色经济，突出发展民营经济，大力发展园区经济，加快推进卫星城建设和城乡一体化步伐，实现县域经济发展的新跨越。三是加快郑汴产业带建设。促进郑州、开封区域协调发展，进一步推进电信同城、金融同城和科技、教育、文化、旅游、广电等资源的整合共享。加快郑汴产业带基础设施建设，结合项目实施推进村庄整合并迁。加大招商引资力度，尽快掀起建设热潮。四是加快郑州航空港区建设。认真落实《郑州国际航空枢纽暨港区建设规划纲要》，研究制定支持航空港区发展的政策措施。加快基础设施建设，加大项目策划和招商引资力度，积极培育主导产业，重点建设航空物流中心，大力发展临空经济。

　　四、推进城乡统筹发展和社会主义新农村建设

　　按照形成城乡一体化新格局的要求，突出加强农业和农村基础设施建设，积极促进农业稳定发展、农民持续增收，努力保障主要农产品基本供给，切实解决农村民生问题，扎实推进新农村建设。一是切实加大“三农”投入力度。今年财政支农投入的增量、固定资产投资用于农村的增量、政府土地出让收入用于农村建设的增量都要明显高于上年。继续实施好各项强农惠农政策，确保补贴资金落实到位。二是大力发展现代农业。稳定粮食播种面积，优化品种结构，实现粮食生产稳定发展。加快实施“菜篮子”工程，确保“菜篮子”产品的有效供应。大力调整农业结构，突出发展优质、高效、专用农作物，逐步建立起各类具有区域化优势的产业化生产基地，支持一批重点龙头企业做大做强。大力发展农民专业合作组织，提高农业组织化程度和服务水平。加强农业科技推广运用，大力培养农村实用人才。加快畜牧业发展步伐，编制完成并组织实施沿黄绿色畜牧产业带发展规划。三是加强农村基础设施建设。在完成“村村通”的基础上，筹措资金８７００万元，新建改建乡村公路５００公里以上。筹措资金８０００万元，解决２０万人的安全饮水问题。筹措资金８０００万元，新增沼气池４万座，积极推进农村改灶、改圈、改厕。完成１３座中小水库除险加固建设任务。认真落实库区移民扶持政策，做好移民安置工作。不断提高扶贫开发水平，全年实现１万人脱贫。四是分类推进新农村建设。继续实施“百村示范、千村整治”工程，突出抓好７０个重点示范村建设。设立１亿元新农村建设引导资金，从环境卫生综合整治入手，因地制宜，分类指导，加快农村饮水、道路、沼气、文化、卫生等基础设施建设，有效改善环境卫生状况和村容村貌，推动社会主义新农村建设取得明显成效。

　　五、不断提高城市建设和管理水平

　　按照中原城市群总体发展规划，努力构建以中心城区和郑东新区为主体、近郊组团为支撑、卫星城为拱卫、重点小城镇为节点的现代化都市圈。一是强化综合交通枢纽地位。把强化综合交通枢纽优势摆上更加突出的位置，以航空港、铁路港、公路港、信息港建设为重点，加快形成多种交通方式综合链接的现代交通体系。围绕中原城市群核心区建设，加快建设郑州到中原城市群各中心城市间的快速通道，着力构建９０分钟经济圈；加快建设中心城区到卫星城的快速通道，着力构建半小时经济圈；加快实施《郑州综合交通近期建设规划》，构筑中心城区“一环两横一纵”的快速道路系统。着力推进一批重大综合交通枢纽工程建设，加快郑州国际航空枢纽、高速铁路客运专线枢纽工程建设，做好郑州－西安客运专线、郑州－武汉客运专线、郑新黄河大桥、连霍高速郑州－洛阳段改扩建、开封－新郑机场高速公路郑州段的配合协调工作，做好郑汴城际轻轨项目前期工作，争取开工建设黄河五桥，积极做好１０７国道东迁的前期工作。二是改造提升中心城区。加快城市基础设施建设步伐，重点实施十项城建工程，即：快速轨道交通工程，陇海路、沙口路高架快速通道工程，解放路跨线立交工程，综合交通枢纽场站工程，火车站西广场工程，火车站地下交通连通工程，中州大道北段工程，西四环工程，西流湖公园工程和郑州植物园工程。同时，建设一批下穿式隧道和人行过街设施，继续实施省行政区道路综合整治，加快推进出入市口、城乡接合部和背街小巷的综合整治。加强道路交通组织和管理，增加公交线路和运营车辆，大力发展公共交通事业。继续加快城中村和旧城、危旧房改造步伐，注重与城市产业发展和主要功能统筹考虑，进一步提高城市的综合承载能力。加强城市绿化美化彩化，市区新增绿地５００万平方米，增加一批市区雕塑作品。三是强化郑东新区产业集聚功能。加快郑东新区的二次招商步伐，由大规模基础设施建设向完善功能、提高品质、聚集人气转变，进一步提高城市管理和社会事务管理水平。积极培育新兴产业，加快发展金融、会展、物流、文化、创意、信息咨询等现代服务业，引进国内外知名金融机构和企业总部入驻，着力打造郑东新区经济发展高地。继续加快建设步伐，年内完成投资１１２亿元，累计完成投资６１８亿元，建成区面积达到６０～７０平方公里，入住人口达到２５万人以上。继续抓好基础设施建设，完善商业网点、教育、医疗、餐饮等配套设施。四是提高城市精细化管理水平。深入开展城市管理年活动，抓好精品街区的综合整治。全面落实城市区域控制性详规，严格按照规划开展建设。继续大力开展违法占地、违法建设集中整治活动。加快数字化城市管理系统建设，提升城市数字化管理水平。五是加快卫星城和小城镇建设。深入贯彻落实《城乡规划法》，高标准编制城乡一体化发展规划，加强指导，强力推进。把卫星城建设作为今年一项重点工作，进一步完善配套设施，加快人口聚集和产业功能培育，着力提高卫星城建设的标准和水平。因地制宜，突出特色，加快小城镇发展步伐。

　　六、继续深化改革扩大开放

　　今年是我国改革开放３０周年，我们要认真总结改革开放以来的历史经验，进一步解放思想，加快改革开放步伐。一是进一步深化国企改革。大力推进国有资本调整和国有企业战略重组，推动国有资本向优势行业和关键领域集中。通过规范上市、中外合资等多种途径完善股权结构，实现投资主体多元化。努力解决国有企业改革的一些遗留问题。二是大力发展非公有制经济。完善促进个体私营经济和中小企业发展的配套政策，加强信用担保、社会化服务等体系建设。鼓励引导非公有制企业加快技术创新，提升产品档次和企业素质。三是深化投融资体制改革。健全投资项目审核制和备案制，落实企业投资自主权。规范政府投资行为，建立政府投资评审机制，全面推行政府项目代建制。完善政府各类投资公司的运行机制，提高资金使用效率和融资水平。加快企业上市步伐，力争新上市企业２～３家。四是推进金融改革。引进战略投资者对市商业银行、百瑞信托、中小企业担保公司实施增资扩股；支持农行完成股改工作；加快推进农村信用社改革，做好组建村镇银行的有关准备工作。同时，全面推进交通、水利、粮食、供销、公用事业和城市管理体制等领域的改革，加快推进集体林权制度改革。

　　继续扩大对外开放。创新招商方式，提高利用外资水平，实施“大招商、招大商”，优化投资结构，重点引进一批优势企业和行业龙头企业，鼓励和促进境内外资本投向高新技术产业、现代服务业、现代农业、公益性基础设施等领域。严格限制引进高耗能、高排放项目。优化投资环境，完善落实外商投资无偿代理制和投资便利化措施。优化进出口商品结构，积极扩大高新技术产品和高附加值产品出口，提升劳动密集型出口产品档次，积极引导加工贸易转型升级。坚持实施“走出去”战略，支持企业开展对外投资和对外承包工程，扩大外派劳务规模。全年实际利用外资达到１３亿美元，增长３０％；引进境内域外资金４００亿元，增长３０％；市属及以下企业出口完成１９．８６亿美元，增长２０％。

　　七、着力加强资源节约和环境保护

　　坚持节约资源和环境保护的基本国策，强化生态文明理念，增强可持续发展能力。一是打好节能减排攻坚战。全面落实《郑州市节能减排实施方案》，按照国家、省节能减排统计监测及考核实施方案的要求，对节能目标完成情况进行量化考核，实行行政问责制和“一票否决”制，确保节能减排目标实现。进一步淘汰落后生产能力，加快落实电力、水泥、钢铁、造纸等行业淘汰落后产能计划，努力完成１４５万千瓦小火电机组的关停任务。严格执行能耗准入标准，突出抓好耗能５０００吨标准煤以上企业的节能工作。继续推进重点节能工程建设，加快节能减排技术进步。继续开展土地“三项整治”，加大违法用地整治力度，对批而不建、长期闲置的土地依法收回。以开发区、工业集聚区为重点，大力建设多层标准厂房。积极推广新型墙材。基本完成煤炭资源整合。加快发展循环经济，力争５家以上县（市）区、园区或企业纳入省一级循环经济试点。二是加强环境综合整治。继续抓好重点流域、区域和行业的环境综合整治。对达不到排放要求的企业实施限期治理、停产治理或强制搬迁。加强燃煤电厂除尘脱硫设施建设和运行监管，各类燃煤机组全部完成脱硫设施建设任务。加强污水处理厂建设。开工建设五龙口污水处理厂二期工程，加快建设陈三桥污水处理厂，完成王新庄污水处理厂脱氮工程建设并投入使用。加强各县（市）污水处理厂配套工程建设，完善环保在线监测体系，提高污水集中处理率。推动污水、垃圾处理产业化，加快发展环保产业。控制大气污染物排放，确保全年市区空气质量二级以上天数达到３００天以上。加大农村环境治理力度，改善农村人居环境。三是抓好生态建设。以森林生态城森林组团建设和通道绿化为重点，加快造林绿化步伐，全年完成造林２６．４万亩，其中，完成森林生态城造林２０．７万亩。继续实施林业生态村绿化工程，建成林业生态模范村５个、林业生态村３２个。加快生态水系建设，开工治理七里河、十七里河、十八里河、魏河，加快贾鲁河、索须河下游段治理，开挖南运河。加快南水北调工程建设。加强湿地保护，加快黄河湿地公园建设。

　　八、积极推动文化大发展大繁荣

　　编制和完善文化产业发展规划，努力建设一批标志性文化基础设施，实施一批重大文化产业项目，培育一批具有较强竞争力的文化企业，创作一批高质量的文化精品，实现由文化资源大市向文化强市的跨越。一是大力发展文化事业。深化文化体制改革，积极推进１４家文化企业单位的改制工作。建立健全公共文化服务体系，开工建设郑州图书新馆和郑州市档案新馆；抓紧做好郑州群艺馆、郑州科技馆新馆和郑州体育馆的前期工作，力争早日开工。加强县（市）区文化馆、图书馆、乡镇文化站和新农村文化大院建设，新建改建５００个农村文化大院，为城市社区文化活动中心和农村文化大院各培训５００名文化艺术骨干，扶持建立１００支社区和农村文化特色队伍。在７００个行政村各建设一个硬化的标准篮球场，在２０户以上已通电自然村实现５万户无线数字电视信号“户户通”。做好嵩山历史建筑群申报世界文化遗产工作。更加重视非物质文化遗产的保护，进一步加大对地方戏曲等优秀传统文化的扶持力度。以迎奥运为契机，普及全民健身运动，提高竞技体育水平。二是加快发展文化旅游产业。不断完善大型舞剧《云水洛神》和《禅宗少林?音乐大典》实景演出；做好小樱桃动漫产业园和快乐星球产业园等项目的前期工作，力争年内开工建设；积极推进郑州印刷包装工业园基础设施建设。加快组建中原传媒集团、郑州新华图书发行集团、快乐星球股份有限公司，推动郑州广电信息网络有限公司上市，培育一批文化产业企业集团。加快旅游产业发展。全面提升嵩山少林、黄帝故里、黄河风景名胜区等精品景区的品位和档次，全面启动商城遗址公园一期工程城墙本体保护工作，高起点规划建设雁鸣湖生态、旅游、休闲景区，编制完成并组织实施沿黄生态文化旅游产业带发展规划，大力发展观光休闲农业和旅游“农家乐”。发展奥运旅游，吸引更多的国内外游客。三是积极建设和谐文化。重视城乡、区域文化协调发展，着力丰富农村、偏远地区、进城务工人员的精神文化生活。加强网络文化建设和管理，营造良好网络环境。大力弘扬爱国主义、集体主义、社会主义思想，加强社会公德、职业道德、家庭美德和个人品德教育。四是大力开展创建全国文明城市工作。组织开展“迎奥运、讲文明、树新风”活动，广泛开展科教、文体、法律、卫生进社区和文化、科技、卫生“三下乡”活动，加强广场文化、社区文化、校园文化建设。积极开展郑州解放６０周年纪念活动，激发全市人民“热爱家乡、增辉郑州”的热情。

　　九、加快推进以改善民生为重点的社会建设

　　着力保障和改善民生，促进社会公平正义，努力使全市人民学有所教、劳有所得、病有所医、老有所养、住有所居。一是认真做好就业再就业和收入分配工作。继续实施积极的就业政策，扩大就业规模。全年城镇新增就业１２万人，及时消除城市“零就业”家庭；完成农村富余劳动力技能培训６万人次，实现农村劳动力转移就业１２万人。积极做好高校毕业生、退伍转业军人、残疾人就业工作。继续深化收入分配制度改革，建立职工工资正常增长机制，逐步提高最低工资标准。认真落实职工带薪休假制度。解决好拖欠职工工资和农民工工资问题。二是优先发展教育。全面实施城乡免费义务教育，从今年秋季起免除城市义务教育阶段学杂费。认真落实《郑州市市区中小学布局规划》，加大中小学校建设力度，年内市区新建改扩建中小学校１５所，不断解决外来人员子女入学和城镇大班额问题。提高农村义务教育阶段中小学公用经费标准，确保小学生人均达到２３０元、初中生人均达到３７５元；提高农村困难家庭寄宿生生活费补助标准，小学、初中由每名学生每天１元分别提高到２元和３元。进一步提高中小学校教师的工资标准和福利待遇。继续推进薄弱中小学改造和农村中小学布局调整，建立农村中小学校舍维修改造长效机制，完成５０所农村初中实验室建设装备任务。加快普及高中阶段教育。大力发展职业教育，加快郑州市职业教育基地建设步伐。不断提高市属高等教育办学层次。鼓励社会力量兴办教育，落实农村民办义务教育学生“两免一补”政策。对我市低保家庭和生活特别困难家庭考上高中（含职业高中和中专）、大学的学生分别给予５０００元、８０００元的一次性资助。重视农村“留守儿童”教育。三是建立基本医疗卫生制度。加快建立覆盖城乡居民的基本医疗卫生保健制度，重点加强农村三级卫生服务网络建设，建成３００个标准化村卫生所，对所有承担公共卫生服务的乡村医生每人每月补助２００元。建立城乡医院对口支援、大医院和社区卫生机构双向转诊、高中级医疗卫生技术人员定期到基层服务制度。加快城市社区卫生服务体系建设，完善社区卫生服务功能，建成中心城区１５分钟社区卫生服务圈，为１万名独居老人安装紧急呼叫装置。推行“片医”负责制，逐步让每一位郑州市民都拥有社区保健医生。加强公共卫生服务体系建设，抓好重大传染病的预防控制工作。加强医疗卫生基础设施建设，开工建设市疾控中心、市妇女儿童医院和市六院呼吸道病房楼。完善大病救助制度，将血友病、白血病、尿毒症等疾病纳入救助范围。对残疾儿童实施康复救助，为全市１万名白内障患者免费实施复明手术。认真实施国家扩大免疫规划，将甲肝、流脑等１５种可以通过接种疫苗有效预防的传染病纳入国家免疫规划范围。大力发展中医药事业，积极推进中西医结合。四是建立覆盖城乡居民的社会保障体系。继续完善城镇职工养老保险制度，从今年起连续３年提高企业退休人员养老金标准。探索城乡居民养老保险试点工作，加快建立覆盖城乡居民的养老保障体系，积极推广城市社区养老模式，新建改扩建２５所农村敬老院，农村五保户集中供养率提高到５０％。努力扩大城镇居民医疗保险参保率，确保城镇居民医保工作规范运行。进一步完善新型农村合作医疗制度，参合标准提高到每人１００元，财政补助标准提高到每人８０元。大力实施农民工“平安计划”，高风险企业农民工基本参加工伤保险。高度重视并切实解决好失地农民的生活保障问题。大力发展残疾人事业、社会福利和慈善事业。深化殡葬改革，年内完成农村１４６个公益性公墓、２９０个骨灰堂的建设任务，加快郑州市殡仪馆搬迁步伐。落实好稳定物价的各项政策措施，加强价格监管，保持市场物价基本稳定；完善对低收入群众补助办法，切实保障困难群众基本生活。加强社会保障性住房建设。编制完成全市低收入家庭住房发展规划，逐步解决低收入家庭住房困难问题。高度重视外来务工人员租房困难问题。力争建成经济适用住房１５０万平方米、廉租房１０万平方米，市区廉租住房覆盖率达到符合条件“双困家庭”的１００％，开工建设周转房３０万平方米。实施农村贫困户危房改造工程。五是完善社会管理。创新社会管理体制机制，发挥各类社会组织的作用，加强社会工作者人才队伍建设。高度重视城市社区管理工作，积极探索社区管理新模式。搞好农村社区建设试点工作，开展第六届村委会换届选举。坚持安全发展，强化安全生产管理和监督，有效遏制重特大安全事故的发生。加强产品质量和食品药品安全监管，努力使城市、农村食品抽检合格率分别提高１个和２个百分点，鼓励药品连锁和集中配送向农村发展。完善信访制度，加强人民调解工作，有效预防和化解各类社会矛盾。健全社会治安防控体系，加强社会治安综合治理，深入开展平安创建活动，做好奥运期间的安保和反恐工作。加强城乡警务工作。全市新建改建标准化警务室１００５个，在５０％以上的社区安装视频监控平台，在６０％以上的行政村安装技防设施。坚持计划生育基本国策，建立完善统筹解决人口问题的长效机制，继续稳定低生育水平，优化人口结构；重视婚前医学检查工作，提高出生人口素质。六是加快建立民生福利评价监测体系，继续为群众办好“十件实事”。

　　深入开展国防教育，增强全民国防意识。支持驻郑解放军和武警部队建设，抓好人民防空和民兵预备役工作。积极开展双拥共建活动，巩固和发展军政军民团结。充分发挥工会、共青团、妇联等人民团体的桥梁纽带作用。全面贯彻党的民族宗教政策，不断巩固平等、团结、互助、和谐的社会主义民族关系，促进宗教和谐。继续做好统计、审计、外事、侨务、对台事务、社会科学、地方志、档案管理、气象和地震监测等工作。

　　十、努力建设人民满意的服务型政府

　　以转变政府职能为核心，以增强政府执行力和公信力为出发点，大力推进体制创新和管理创新，努力构建社会服务体系，建设人民满意的服务型政府。一是转变政府职能。在加强和改善经济调节和市场监管的同时，更加注重履行社会管理和公共服务职能，加快公共教育、公共卫生和基本医疗服务、公共文化服务、就业服务、社会保障、公共安全等服务体系建设。认真落实政府信息公开条例，进一步加强电子政务建设，保障人民群众的知情权、参与权和监督权。二是严格依法行政。自觉接受人大及其常委会的法律监督和政协的民主监督，严格执行人大及其常委会的决议、决定，广泛听取各民主党派、工商联和无党派人士的意见和建议。加快我市社会管理和公共服务等领域的立法步伐，提高立法质量。认真落实今年新实施的劳动合同法、就业促进法等一批法律法规，依法规范行政行为，形成自觉学法守法用法的良好氛围。加大《郑州市城市养犬管理条例》等地方性法规的执行力度。健全质询、问责、经济责任审计、引咎辞职等制度，确保权力在阳光下运行。三是加强廉政建设。牢记“两个务必”，厉行节约，勤俭办事，严格财政支出管理，严格控制修建楼堂馆所，严格公费出国出境管理，严格规范公务接待行为，坚决反对铺张浪费和奢靡之风，建设节约型机关。加强反腐倡廉建设，坚决惩治和有效预防腐败。四是改进工作作风。牢记全心全意为人民服务的宗旨，倾听群众呼声，了解群众愿望，多为群众办好事、办实事。精简会议和文件，减少检查评比活动，进一步提高工作实效。大兴调查研究之风，深入基层，深入群众，掌握真实情况，解决实际问题。树立正确的政绩观，建立健全符合科学发展观要求的政府工作考核体系，加强目标管理，推行行政问责和政府绩效管理制度，加大督察力度，确保工作落实。

　　各位代表，建设全国区域性中心城市，建成全省的首善之区，我们还需要付出很大的努力；领跑中原崛起，促进中部崛起，我们使命光荣，任重道远。让我们以党的十七大精神为指导，高举中国特色社会主义伟大旗帜，深入贯彻落实科学发展观，紧密团结在以胡锦涛同志为总书记的党中央周围，在省委、省政府和市委的正确领导下，团结拼搏，奋勇争先，为完成本届政府任期各项目标任务，为实现经济社会跨越式发展，为夺取全面建设小康社会新胜利而努力奋斗！
